# PCSK9 as a Potential Regulator of Endothelial Dysfunction: Mechanistic Insights and Future Directions

**DOI:** 10.3390/ijms27146473

**Published:** 2026-07-21

**Authors:** Anisa Qisti Mathriul, Maharani Hestu Mukti Wisesa, Vicko Suswidiantoro, Meidi Utami Puteri, Mitsuyasu Kato, Fadlina Chany Saputri

**Affiliations:** 1 Faculty of Pharmacy, Universitas Indonesia, UI Depok Campus, Depok 16424, West Java, Indonesia; anisa.qisti@ui.ac.id (A.Q.M.); maharani.hestu@ui.ac.id (M.H.M.W.); vicko.suswidiantoro@ui.ac.id (V.S.); mit-kato@md.tsukuba.ac.jp (M.K.); 2 Laboratory of Pharmacology and Toxicology, Pharmacy Department, Faculty of Health, Universitas Aisyah Pringsewu, Pringsewu 35372, Lampung, Indonesia; 3 Laboratory of Pharmacology and Toxicology, Faculty of Pharmacy, Universitas Indonesia, Kampus UI, Depok 16424, West Java, Indonesia; meidiutami@farmasi.ui.ac.id; 4 National Metabolomics Collaborative Research Center, Faculty of Pharmacy, Universitas Indonesia, Kampus UI, Depok 16424, West Java, Indonesia; 5 Department of Experimental Pathology, Faculty of Medicine, University of Tsukuba, Tsukuba 305-8575, Ibaraki, Japan

**Keywords:** PCSK9, endothelial dysfunction, vascular inflammation, oxidative stress, experimental and clinical evidence

## Abstract

The discovery of proprotein convertase subtilisin/kexin type 9 (PCSK9) and its role in promoting the degradation of the low-density lipoprotein receptor (LDLR) has revolutionized lipid-lowering therapy, offering new therapy for patients with hypercholesterolemia. Notably, PCSK9 is now being explored for functions beyond lipid regulation. Emerging evidence implicates PCSK9 in vascular pathology by influencing endothelial dysfunction, inflammatory signaling, oxidative stress, and vascular homeostasis—potentially through LDLR-independent mechanisms. This review critically evaluates PCSK9’s potential as a regulator of endothelial dysfunction, synthesizing findings from *in vitro*, *in vivo*, and clinical studies. While current PCSK9-targeted therapies, including monoclonal antibodies and siRNA-based agents, have demonstrated robust efficacy in lowering lipid levels and reducing cardiovascular risk, their direct effects on endothelial PCSK9 signaling remain unclear. On the other hand, novel approaches—such as peptide-based inhibitors, intracellular modulation, and natural compounds—resulted in new insights into endothelial-specific PCSK9 pathways. Overall, this review highlights the need to bridge experimental and clinical evidence by applying endothelial-focused models, multi-omics analyses, and functional vascular assessments to clarify the role of PCSK9 in endothelial dysfunction and to propose future strategies for cardiovascular interventions targeting PCSK9-mediated endothelial dysfunction.

## 1. Introduction

Endothelial dysfunction is widely recognized as an early and pivotal event in the onset and progression of atherosclerotic cardiovascular disease [1,2], arising from a complex interaction between metabolic disturbance and vascular inflammation [3]. Under normal physiological conditions, endothelial cells maintain vascular homeostasis by regulating vasomotor tone, preserving barrier integrity, sustaining haemostatic balance, and modulating immune responses [4]. When these tightly controlled processes become disrupted, nitric oxide bioavailability declines, oxidative stress is amplified, and pro-inflammatory signalling pathways are activated. Collectively, these alterations promote leukocyte adhesion, increase endothelial permeability, and contribute to plaque formation [5].

Although dyslipidemia has long been regarded as a primary driver of endothelial injury, growing evidence suggests that lipid-independent pathways also play a substantial role in vascular dysfunction. In this regard, proprotein convertase subtilisin/kexin type 9 (PCSK9) has gained considerable attention. Originally characterized as a hepatic regulator responsible for low-density lipoprotein receptor (LDLR) degradation, PCSK9 is now known to be expressed in multiple vascular cell types, particularly under inflammatory conditions [6,7,8].

PCSK9, a serine protease, is well established as a key regulator of lipid metabolism through its promotion of LDLR degradation in hepatocytes [9]. However, its biological functions extend beyond lipid regulation [10]. Evidence indicates that PCSK9 is present within human atherosclerotic plaques and is expressed in both vascular smooth muscle cells and endothelial cells following exposure to inflammatory stimuli [11]. Experimental studies suggest that PCSK9 overexpression may be associated with endothelial apoptosis and accelerated atheroma development in model systems [12]. Conversely, PCSK9 deficiency or inhibition has been associated with reduced plaque progression in experimental settings, accompanied by decreased expression of lectin-like oxidized LDL receptor-1 (LOX-1) and TNF Receptor Associated Factor 6 (TRAF6) in endothelial cells [13]. Clinical observations further support this association; for instance, in patients with ST-elevation myocardial infarction (STEMI), lower circulating PCSK9 levels correlate with reduced endothelial apoptosis, highlighting its potential involvement in endothelial dysfunction during acute coronary events [14]. Taken together, these findings support a potential contribution of PCSK9 to endothelial dysfunction and suggest that it may represent a therapeutic target in atherosclerosis. Monoclonal antibodies, including alirocumab and evolocumab, as well as small interfering RNA (siRNA)-based therapies such as inclisiran, have demonstrated substantial efficacy in lowering LDL-C levels and reducing adverse cardiovascular outcomes. Importantly, beyond their lipid-lowering effects, PCSK9 inhibitors have also been associated with improvements in endothelial function, although the extent to which these effects are direct remains uncertain. Meanwhile, emerging therapeutic approaches, including small-molecule inhibitors, remain under development, and their impact on endothelial dysfunction has yet to be fully elucidated. This review therefore aims to critically examine the proposed mechanistic roles of PCSK9 in endothelial dysfunction and to evaluate both established and emerging PCSK9-targeted therapies as potential strategies for vascular protection.

Therefore, throughout this review, proposed PCSK9-related pathways are interpreted as biologically plausible and hypothesis-generating unless they have been directly validated in relevant human vascular systems or clinical interventional studies.

## 2. Methodology of Literature Review

This article is presented as a narrative review intended to provide a critical synthesis of current evidence regarding the lipid-independent role of proprotein convertase subtilisin/kexin type 9 (PCSK9) in endothelial dysfunction and its therapeutic implications. Relevant literature was identified through comprehensive searches of PubMed, Scopus, and Web of Science up to June 2025 using combinations of the keywords “PCSK9”, “endothelial dysfunction”, “atherosclerosis”, “vascular inflammation”, “oxidative stress”, “LOX-1”, “TLR4”, “pyroptosis”, “efferocytosis”, “PCSK9 inhibitors”, and “endothelial cells”. Only peer-reviewed articles published in English were considered. Priority was given to mechanistic *in vitro* studies, animal experiments, translational investigations, and clinical studies directly addressing endothelial biology or vascular effects of PCSK9 modulation. Additional relevant publications were identified through manual screening of reference lists. Owing to the heterogeneous nature of the available evidence and the objective of providing a critical mechanistic perspective, a narrative review methodology was considered more appropriate than a systematic review. Throughout the review, evidence derived from *in vitro* experiments, animal models, and clinical studies is explicitly distinguished to facilitate interpretation of the current level of scientific evidence supporting each proposed mechanism and therapeutic strategy.

## 3. Molecular Pathways Associated with PCSK9-Related Endothelial Cell Dysfunction

### 3.1. Apoptosis and Pyroptosis

Growing evidence suggests that PCSK9 may be associated with endothelial injury and may contribute to endothelial dysfunction through multiple programmed cell death pathways, including apoptosis and pyroptosis [15,16,17,18,19]. In experimental studies, stress inducers such as oxidized low-density lipoprotein (ox-LDL) have been observed to upregulate PCSK9 expression in human umbilical vein endothelial cells (HUVECs), a process that positively correlates with increased rates of cellular damage and programmed cell death [15,16].

Research by Wu et al. suggests that this endothelial injury involves the intrinsic apoptotic cascade, demonstrating a concentration-dependent relationship between PCSK9 expression and a reduced Bcl-2/Bax ratio [15]. This shift is thought to facilitate the activation of the initiator caspase-9 and the executioner caspase-3, leading to the condensation of nuclear chromatin and cell shrinkage [15]. Complementing these findings, Li et al. reported that ox-LDL-induced PCSK9 expression may also contribute to apoptosis through the mitogen-activated protein kinase (MAPK) signaling cascade [16]. Their data indicate that silencing PCSK9 expression is associated with a reduction in the phosphorylation of p38 and JNK, suggesting that the JNK/p38 MAPK-dependent pathway may be a potential mediator of endothelial cell death in atherosclerosis [16]. Collectively, experimental data from siRNA and shRNA-mediated silencing indicate that reducing PCSK9 levels can mitigate these apoptotic effects, potentially by restoring the balance between pro-apoptotic and anti-apoptotic factors and inhibiting the activation of the caspase and MAPK cascades [16]. While it is still under investigation whether PCSK9 acts directly on these apoptotic-related proteins or triggers the cascade via alternative indirect pathways, evidence from previous studies examining Bax levels suggests possible involvement of mitochondrial apoptotic pathways [20].

Related to these findings, PCSK9 has also been increasingly implicated in pyroptosis—a distinct form of programmed cell death that is dependent on caspase-1 activation [17,18,19]. Mechanistic studies propose that PCSK9 may promote pyroptosis, potentially through inhibition of UQCRC1, a key subunit of the mitochondrial respiratory chain [19]. This inhibition contributes to mitochondrial dysfunction, characterized by the loss of membrane potential and increased production of reactive oxygen species (ROS) [19]. Elevated ROS subsequently activate the NLRP3 inflammasome, which, through recruitment of the adapter protein ASC, leads to caspase-1 activation [19]. This proposed cascade may result in the release of inflammatory cytokines such as IL-1β and IL-18, as well as the formation of gasdermin-D pores in the cell membrane, driving the inflammatory cell death process [19].

Furthermore, supporting the possible involvement of mitochondrial dysfunction in PCSK9-associated endothelial dysfunction, Martino et al. investigated the molecular mechanisms underlying PCSK9-induced endothelial cell damage [18]. Their research identified a close association between elevated PCSK9 levels and dysregulation of the miR-15b-5p–SIRT4 axis, a molecular pathway essential for mitochondrial integrity and cellular protection [18]. Increased expression of PCSK9 was associated with downregulation of SIRT4, a mitochondrial protein with anti-inflammatory and anti-apoptotic properties [18]. Collectively, these findings highlight a possible role of the miR-15b-5p–SIRT4 axis in PCSK9-associated endothelial injury and suggest that targeting PCSK9 and mitochondrial function may offer promising therapeutic strategies to counteract vascular inflammation and cell death [18].

**Figure 1:**
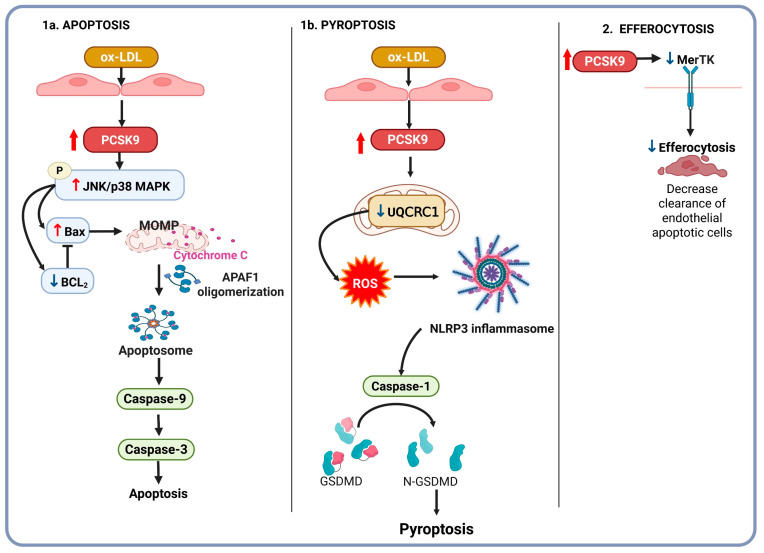
Proposed PCSK9-associated mitochondrial apoptotic and efferocytosis-related pathways in endothelial cells. (1a) Apoptosis: Upstream stimuli, including ox-LDL, upregulate PCSK9 expression in endothelial cells. Elevated PCSK9 may increase phosphorylated JNK/p38 MAPK, resulting in downregulation of Bcl-2 and upregulation of Bax. Elevated Bax, leading to cytochrome c release and activation of caspase-9 and caspase-3 thus initiating endothelial apoptosis. (1b) Pyroptosis: In parallel, PCSK9 downregulates UQCRC1, resulting in increased mitochondrial ROS production. Elevated ROS activates NLRP3 inflammasome, which in turn activates caspase-1, leading to gasdermin-D cleavage and pore formation in the cell membrane, resulting in pyroptosis. (2) Efferocytosis: Furthermore, PCSK9 has been proposed to impair efferocytosis by downregulating MerTK expression, leading to defective clearance and accumulation of apoptotic cells. Created in Biorender. Hestu, M. (2026) https://BioRender.com/tt3nn3q.

Both apoptosis and pyroptosis of endothelial cells are important contributors to endothelial dysfunction [21,22]. Apoptosis disrupts the integrity of the endothelial monolayer, leading to increased vascular permeability and enhanced monocyte adhesion, thereby aggravating vascular inflammation and accelerating atherosclerotic plaque formation [21]. Similarly, pyroptosis, a highly inflammatory form of programmed cell death, further disrupts endothelial homeostasis by promoting the release of pro-inflammatory cytokines and exacerbating vascular injury [22]. Together, these PCSK9-associated cell death pathways may contribute to endothelial dysfunction and atherosclerosis progression, although their causal contribution in humans requires further validation. As shown in Figure 1 the PCSK9-associated mitochondrial apoptotic and pyroptotic pathways in endothelial cells are illustrated.

### 3.2. Efferocytosis

Next, in addition to its proposed associations with endothelial cell apoptosis and pyroptosis, initial evidence suggests that PCSK9 may attenuate efferocytosis—the process by which cells clear apoptotic debris [23,24,25]. Current evidence supporting the involvement of PCSK9 in endothelial efferocytosis remains limited and originates primarily from mechanistic studies conducted in cultured endothelial cells and experimental animal models. Whether impaired efferocytosis contributes directly to human endothelial dysfunction through PCSK9 signalling has not yet been confirmed clinically. Accordingly, the mechanisms described below should be interpreted as emerging biological hypotheses rather than clinically established pathways.

A study by Liu et al. using primary human and mouse aortic endothelial cells argues for a role of PCSK9 in attenuating endothelial cell efferocytosis, suggesting a mechanistic link between PCSK9-mediated endothelial dysfunction and vascular aging [24]. The underlying molecular mechanism shown by their study is that PCSK9 acts as a negative regulator of MerTK, a critical receptor that enables endothelial cells to recognize and clear apoptotic cell debris. The study also reported that overexpression of PCSK9, achieved by administering recombinant PCSK9 protein, was observed to potentially downregulate MerTK expression in a dose-dependent manner, thereby suggesting a decrease in efferocytosis capacity [24]. Consequently, if PCSK9-dependent impairment of efferocytosis occurs, apoptotic cells may accumulate, leading to increased oxidative stress (ROS), activation of pro-inflammatory pathways (MAPK and NF-κB), and accelerated vascular aging. Importantly, they also showed that inhibiting PCSK9—either pharmacologically or through genetic deletion—can restore MerTK expression and improve efferocytosis capacity in aged mice [24].

These phenomena are supported by other studies focusing on the roles of MerTK independently of PCSK9 [23,24,25]. Li et al. reported that MerTK is essential for maintaining the endothelial normal physiological functions, as it mediates cytoskeletal reorganization and facilitates the uptake of apoptotic cells [25]. In short, their findings demonstrate that MerTK is essential for vascular and immune health by facilitating the clearance of apoptotic cell debris [23,25]. Thus, PCSK9 is proposed to disrupt this protective process by negatively regulating MerTK, thereby reducing efferocytosis and leading to persistent inflammation and accelerated vascular aging [24]. Taken together, these observations suggest that impaired efferocytosis may contribute to endothelial dysfunction, but the causal contribution of PCSK9 requires further validation.

### 3.3. Inflammation

In addition to studies on PCSK9-associated apoptosis, pyroptosis, and reduced efferocytosis—processes that may contribute to endothelial damage and dysfunction—emerging evidence suggests that PCSK9 may also contribute to endothelial dysfunction by modulating inflammatory pathways [26].

Although several experimental studies have reported potential associations between PCSK9 and various inflammatory mediators, the evidence is still fragmented and predominantly derived from specific *in vitro* or animal models. Instead of a comprehensive network, the following sections outline proposed pathways involving TLR4, LOX-1, and SIRT3 that may contribute to PCSK9-mediated endothelial dysfunction [27,28,29]. Modulation of these proteins by PCSK9 has been associated with inflammatory pathway activation, pro-inflammatory cytokine production, and adhesion molecule expression, which may contribute to endothelial dysfunction and atherosclerosis progression [27,28,29]. To better understand how PCSK9 modulates inflammation in endothelial cells through the mentioned proteins, here we review and explain the mechanisms by which PCSK9 regulates each one.

Several studies have reported potential positive feedback crosstalk between PCSK9 and LOX-1 within inflammatory microenvironments, in which each protein can induce the expression of the other [28,29]. LOX-1, a type II transmembrane receptor with a C-type lectin-like extracellular domain, is expressed in endothelial cells, macrophages, and vascular smooth muscle cells [30,31]. Upon activation by ox-LDL, LOX-1 induces endothelial dysfunction through increased production of reactive ROS, which leads to NF-κB activation and result in upregulation of adhesion molecules and enhanced endothelial apoptosis [32]. PCSK9 has been proposed to amplify this process, as elevated PCSK9 expression is associated with increased LOX-1 levels [28,29]. Experimental systems included parallel-plate flow chambers to simulate hemodynamic forces and C57BL/6 mice with PCSK9 or NADPH oxidase subunit knockouts [29,33]. The results suggest that PCSK9 upregulates LOX-1 expression and establishes reciprocal cross-talk with the LOX-1 receptor, with both proteins mutually enhancing each other’s expression, especially under inflammatory stimuli such as lipopolysaccharide (LPS) exposure or low shear stress [29,33]. Mechanistically, the interaction between PCSK9 and LOX-1 has been linked to mitochondrial ROS (mtROS) generation leading to NF-κB pathway activation which results in increased expression of adhesion molecules such as vascular cell adhesion molecule (VCAM-1), monocyte chemoattractant protein-1 (MCP-1), and intercellular adhesion molecule-1 (ICAM-1) [29].

Not only LOX-1, but another protein involved is TLR4 [30,31]. Studies investigating the role of PCSK9-TLR4 in endothelial cell inflammatory signaling have utilized both *in vitro* and *in vivo* models [27,34]. Studies demonstrate that LPS modulates TLR4-mediated inflammatory signaling, thereby activating the NF-κB pathway and increase PCSK9 expression [34]. Subsequent activation of NF-κB promotes the expression of pro-inflammatory cytokines, including IL-1β, IL-6, and TNF-α, as well as adhesion molecules such as VCAM-1, ICAM-1, and E-selectin, which may facilitate monocyte recruitment and vascular inflammation [27,34]. In line with these studies, research utilizing HUVECs and mouse models has shown that PCSK9 suppresses the expression of endothelial nitric oxide synthase (eNOS) and vascular endothelial (VE) cadherin. This suppression, believed to be mediated by the TLR4/MyD88/NF-κB axis, may contribute to endothelial dysfunction and atherosclerosis progression [27,34].

While the roles of PCSK9-LOX-1 and PCSK9-TLR4 interactions in mediating endothelial inflammation are well introduced, recent research suggests that additional molecules may also contribute to PCSK9-associated inflammatory responses. Notably, emerging experimental evidence in human aortic endothelial cells (TeloHAEC) indicates that the beneficial effects of PCSK9 inhibitors—including their anti-inflammatory, anti-autophagic, and antioxidant actions—may be mediated, at least in part, by SIRT3 [35]. SIRT3 is a mitochondrial NAD^+^-dependent deacetylase and a key regulator of mitochondrial protein acetylation [36]. Studies have identified SIRT3 as a crucial mediator of the pleiotropic effects of PCSK9 inhibitors (PCSK9i) in vascular endothelial cells [35]. Specifically, research in TeloHAECs demonstrates that pro-inflammatory stimuli such as IL-6 cause a significant increase in PCSK9 expression and a concurrent decrease in SIRT3 levels. This imbalance is associated with heightened inflammasome activation (NLRP3, caspase-1, IL-1β), increased mitochondrial ROS accumulation, and greater autophagy. Treatment with the PCSK9 inhibitor evolocumab reverses these unwanted changes by restoring SIRT3 expression. Importantly, when SIRT3 is silenced using siRNA, the protective effects of PCSK9 inhibition against IL-6-induced mitochondrial and inflammatory damage are lost, indicating that SIRT3 may be necessary for these non-lipid-lowering, antioxidant, and anti-inflammatory effects of PCSK9i in this experimental context [35]. This molecular relationship is further supported by clinical data from human carotid plaque specimens (*n* = 277), where higher PCSK9 expression correlates with lower SIRT3 levels and higher markers of inflammation (IL-6, IL-1β) and autophagy (LC3B II/I). These findings suggest that PCSK9 inhibitors may help attenuate endothelial dysfunction associated with low-grade inflammation by maintaining SIRT3 expression and its protective effects. However, further detailed mechanistic studies are necessary to fully elucidate the molecular interactions between PCSK9 and SIRT3 [35].

Collectively, available evidence suggests that PCSK9 may be involved in lipid-derived and inflammatory signals through NF-κB activation, oxidative stress, and NLRP3 inflammasome assembly. These pathways should be interpreted as proposed mechanisms rather than as a fully established causal framework for endothelial dysfunction. However, it is important to note that much of the current evidence is derived from *in vitro* and animal studies, and the specific causal role of PCSK9 in human endothelial cells has yet to be fully established. Table 1 summarises the current hierarchy and strength of evidence supporting the proposed molecular mechanisms and therapeutic strategies associated with PCSK9-mediated endothelial dysfunction. The available evidence is categorized according to findings derived from *in vitro* studies, experimental animal models, and human clinical investigations, thereby facilitating interpretation of the current level of evidence and the translational relevance of each proposed mechanism and therapeutic strategy. To distinguish between clinically established interventions and emerging experimental approaches, Table 2 summarises clinically validated PCSK9-targeted therapies, whereas Table 3 outlines novel preclinical strategies currently under investigation.

**Figure 2:**
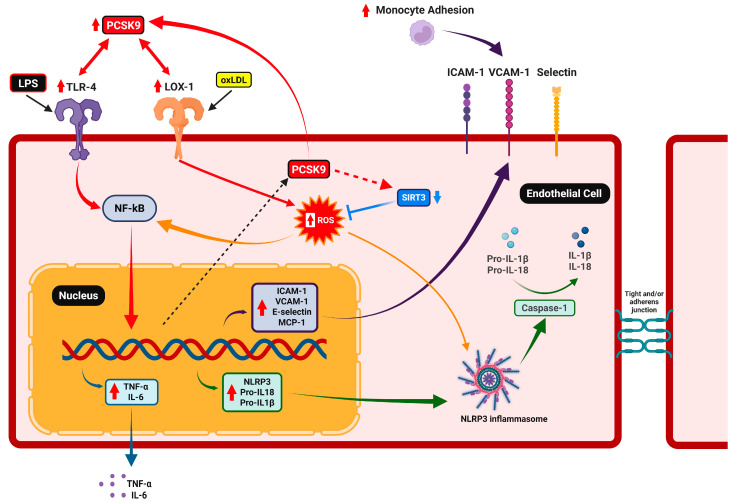
Proposed PCSK9-associated endothelial inflammation and inflammasome activation. Elevated PCSK9 is proposed to amplify endothelial dysfunction through inflammatory and oxidative pathways. NF-κB activation can be initiated either by LPS via TLR4 signaling or by oxLDL through LOX-1-mediated ROS production. This activation cascades into the upregulation of pro-inflammatory genes, including NLRP3, pro-interleukin (IL)-1β, pro-IL-18, tumour necrosis factor-α (TNF-α), and adhesion molecules such as VCAM-1, ICAM-1, E-selectin and PCSK9. Simultaneously, elevated PCSK9 correlated with decreased SIRT3, thereby sustaining high ROS levels. ROS may act as a secondary signal for NLRP3 inflammasome activation. The assembly of the NLRP3 inflammasome leads to caspase-1 activation and subsequent cleavage of pro-IL-1β and pro-IL-18 into their mature forms. Taken together, the subsequently expressed PCSK9 protein promotes a positive feedback loop by mediating crosstalk between the TLR4 and LOX-1 pathway, alongside elevated expression of pro-inflammatory cytokines such as TNF-α and IL-6, which may further amplify endothelial inflammation and monocyte recruitment. Dashed arrow indicates hypothetical pathways. Bidirectional arrow indicates crosstalk. Created in Biorender. Hestu, M. (2026) https://BioRender.com/tt3nn3q.

As illustrated in Figure 2, PCSK9 is proposed to participate in lipid-derived and inflammatory signalling pathways involving NF-κB activation, oxidative stress, and NLRP3 inflammasome assembly.

## 4. PCSK9 Inhibitors as Therapeutic Strategies for Targeting PCSK9 Pathway-Mediated Endothelial Dysfunction

PCSK9-targeted therapies have expanded considerably, ranging from clinically approved agents such as monoclonal antibodies and siRNA-based therapies to emerging experimental approaches, including ATTECs, peptide-based inhibitors, and natural compounds [37,38,39,40,41,42]. While approved therapies have strong clinical evidence for LDL-C reduction and cardiovascular risk lowering, their direct endothelial-specific benefits remain less clearly established. In contrast, newer strategies show mechanistic promise in modulating PCSK9-related endothelial inflammation, oxidative stress, and vascular dysfunction, but most evidence remains limited to preclinical, *in vitro*, or computational studies [36,37,38,39,40]. The following sections discuss each therapeutic approach according to its mechanism of PCSK9 modulation and current level of evidence.

### 4.1. PCSK9 Monoclonal Antibody

Alirocumab and evolocumab are monoclonal antibodies approved by the FDA and EMA for PCSK9 inhibition. By blocking PCSK9, these agents increase hepatic LDLR availability, thereby enhancing LDL-C clearance and substantially reducing plasma LDL-C levels [43]. Beyond their established lipid-lowering effects, accumulating evidence suggests that monoclonal antibodies may also exert vascular protective effects, particularly by attenuating endothelial inflammation and dysfunction.

At the cellular level, PCSK9 inhibition has been shown to suppress inflammatory signaling pathways in endothelial cells. Mechanistic studies indicate that evolocumab reduces the expression of NLRP3 inflammasome components and pro-inflammatory cytokines, including TNF-α, IL-1β, and IL-18, through modulation of the TLR4/MyD88/NF-κB signaling pathway [34]. These findings suggest that PCSK9 inhibition may directly interfere with inflammatory cascades that contribute to endothelial dysfunction.

Consistent with these mechanistic observations, preclinical studies provide further evidence of endothelial protection. In hyperlipidemic APOE*3Leiden.CETP mice, anti-PCSK9 antibody therapy significantly downregulated endothelial adhesion molecules, including ICAM-1 [44]. Because these molecules facilitate monocyte adhesion and infiltration into the vascular wall, their reduction may help limit early inflammatory events involved in atherosclerotic plaque development and progression. In the same experimental context, anti-PCSK9 antibody treatment also decreased TNF-α and IL-6, further supporting its anti-inflammatory effects in the vascular endothelium [44].

Clinical findings also support a potential endothelial impact of PCSK9 monoclonal antibodies in humans. In patients with acute coronary syndrome, administration of evolocumab at a therapeutic dose of 420 mg was associated with reduced colocalization of PCSK9 within endothelial cells and favorable changes in biomarkers related to endothelial dysfunction [45]. These observations suggest that the endothelial relevance of PCSK9 inhibition may extend beyond experimental models.

Collectively, available evidence indicates that alirocumab and evolocumab not only provide robust systemic LDL-C reduction but may also attenuate endothelial inflammatory responses. However, whether these vascular benefits arise from direct modulation of endothelial PCSK9 or occur primarily as secondary consequences of systemic LDL-C lowering remains to be clarified.

### 4.2. Inclisiran

Inclisiran is a small interfering RNA (siRNA)-based therapy designed to target hepatic PCSK9 mRNA. To enable selective uptake by hepatocytes, inclisiran is conjugated with a trivalent N-acetylgalactosamine (GalNAc) ligand, which binds with high affinity to the hepatocyte-specific asialoglycoprotein receptor (ASGPR) [46,47]. After internalization, inclisiran is incorporated into the RNA-induced silencing complex (RISC), where it guides the catalytic cleavage of PCSK9 mRNA [48]. This targeted gene-silencing mechanism suppresses hepatic PCSK9 synthesis, thereby promoting LDLR recycling to the hepatocyte surface and enhancing plasma LDL-C clearance [39,47].

Beyond its established hepatic mechanism, preclinical evidence suggests that inclisiran may also confer vascular protective effects. In an *in vivo* murine model of atherosclerosis, inclisiran administration was reported to suppress endothelial cell pyroptosis within the aortic wall [17]. This effect was accompanied by reduced expression of key pyroptosis-related markers, including NLRP3, ASC, and gasdermin D (GSDMD), at both the gene and protein levels. Consequently, the expression of downstream pro-inflammatory cytokines, such as IL-1β and IL-18, was also attenuated [17]. These findings suggest that inclisiran may influence inflammatory cell-death pathways involved in endothelial dysfunction.

Clinically, inclisiran has demonstrated robust lipid-lowering efficacy, consistently reducing circulating LDL-C levels by approximately 50% in patients with elevated cholesterol levels or high cardiovascular risk [39,47]. However, although its systemic LDL-C-lowering effect is well established in human studies, direct evidence confirming endothelial-specific therapeutic effects in clinical settings remains limited. Therefore, further translational and clinical investigations are needed to determine whether the endothelial protection observed in preclinical models contributes directly to the cardiovascular benefits associated with inclisiran therapy.

### 4.3. Autophagy-Tethering Compound

Autophagy-tethering compounds (ATTECs) are an emerging class of small molecules designed to promote selective degradation of intracellular proteins through the autophagy–lysosome pathway. These compounds act by simultaneously binding a target protein and LC3, a key protein associated with the autophagosome membrane [49]. LC3 exists in two main forms, LC3-I and LC3-II, with LC3-II generated through lipidation of LC3-I by conjugation with phosphatidylethanolamine. This lipidation enables LC3-II to stably associate with autophagosomal membranes. By tethering the target protein to LC3, ATTECs facilitate its recruitment to autophagosomes and subsequent degradation through the autophagolysosomal pathway.

Recently, ATTEC-based approaches have been explored for the targeted degradation of intracellular PCSK9. In cellular models, the ATTEC molecule W6 has shown PCSK9-degrading activity. Suppression of endothelial PCSK9 by W6 was associated with reduced expression of key adhesion molecules, including ICAM-1 and VCAM-1, both of which contribute to endothelial inflammation and atherogenesis [50]. These findings suggest that PCSK9-targeted ATTECs may have potential relevance in modulating endothelial inflammatory responses.

Preclinical *in vivo* evidence further supports the therapeutic potential of this approach. In animal studies, oral administration of the PCSK9-directed ATTEC molecule OY3 at 15 mg/kg/day for 21 days reduced plasma LDL levels and ameliorated atherosclerotic lesions [40]. These findings indicate that targeted degradation of PCSK9 may provide both lipid-lowering and vascular protective effects in experimental models.

Despite these promising mechanistic and preclinical findings, PCSK9-targeted ATTECs remain at an early experimental stage. Current evidence is limited to *in vitro* and animal studies, and their safety, efficacy, and endothelial-specific protective effects have not yet been evaluated in human clinical settings. Further translational studies are therefore required before this strategy can be considered for clinical application.

### 4.4. Peptide-Based Inhibitors

Peptide-based PCSK9 inhibitors are designed to disrupt the interaction between PCSK9 and LDLR by mimicking the EGF(A) domain of LDLR. By occupying the PCSK9-binding interface, these peptides prevent PCSK9-mediated LDLR internalization and lysosomal degradation, thereby preserving LDLR expression on the cell surface and supporting LDL-C clearance [41,51,52]. Pep2-8 is a short synthetic peptide that inhibits PCSK9 binding to LDLR, with an IC50 of 0.8 µM for LDLR and 0.4 µM for the isolated EGF(A) domain [52]. Subsequent structural optimization improved its potency. For example, Fusion-1, generated by linking a groove-binding peptide to Pep2-8 through a GSC linker, restored up to 90% of cell-surface LDLR in HepG2 cells at 5.0 µM and was approximately 20-fold more potent than Pep2-8 [41,51]. In addition, substitution of tyrosine at position 9 with alanine produced a Pep2-8 variant with approximately 50-fold greater potency than the parent peptide [41].

Beyond LDLR preservation, Pep2-8 may also exert vascular protective effects. In a mouse model, Pep2-8 administration reduced NOX4, MAPK, and NF-κB pathway activation, thereby attenuating oxidative stress and inflammatory signaling [24]. It also increased eNOS expression, suggesting improved endothelial function, and upregulated MerTK, which may support vascular repair through enhanced efferocytosis of apoptotic cells [24]. Overall, peptide-based inhibitors provide proof-of-concept that the PCSK9–LDLR interaction can be selectively disrupted using small, structure-guided molecules. However, their clinical relevance remains uncertain because current evidence is limited to biochemical, cellular, and animal studies. Further validation is required to determine whether these endothelial-protective effects can be translated into human clinical benefit.

### 4.5. Natural Compounds

#### 4.5.1. Brazilin

Brazilin, a flavonoid compound derived from *Caesalpinia sappan*, has been identified as a potential PCSK9 inhibitor by disrupting the PCSK9–LDLR interaction at the EGF-A binding site, as shown in in silico and non-cellular *in vitro* models [53]. Beyond its lipid-regulating potential, brazilin also exhibits anti-inflammatory activity, which may be particularly relevant for endothelial dysfunction—a condition in which both PCSK9 signaling and inflammation play critical roles. Notably, studies using macrophage cell lines have shown that brazilin downregulates TLR4 and NF-κB expression, thereby exerting anti-inflammatory effects [54]. Although direct studies on its impact on endothelial cells or its ability to mitigate PCSK9-mediated endothelial dysfunction are limited [6], brazilin’s documented bioactivity offers a strong mechanistic rationale for its endothelial protective potential. Evidence suggests that, during endothelial dysfunction, PCSK9-mediated TLR4 activation triggers downstream inflammatory cascades, including the induction of NLRP3 inflammasome components and pro-inflammatory cytokines such as IL-6 and TNF-α [27,34,55,56,57,58]. Brazilin’s established ability to suppress these same pro-inflammatory mediators, combined with its PCSK9 inhibitory activity, supports its proposed therapeutic value. Accordingly, while direct cell-specific validation is strictly required, the collective data highlight brazilin as a candidate for initial investigation in endothelial models to bridge the current translational gap.

#### 4.5.2. Ginkgolide B

Ginkgolide B is a diterpene trilactone compound derived from *Ginkgo biloba*. In ox-LDL-stimulated HUVECs, Ginkgolide B has been shown to suppress PCSK9 expression at both mRNA and protein levels, with reductions of approximately 30–60% after treatment with 47-236 µM for 24 h [59]. This effect is associated with reduced SREBP-2 expression, suggesting modulation of a key transcriptional regulator of PCSK9 [59]. In silico analyses further suggest that Ginkgolide B may interfere with the PCSK9–LDLR interaction. Beyond PCSK9 regulation, Ginkgolide B also attenuates endothelial oxidative stress and inflammation. Treatment with 59-471 µM for 24 h reduced LOX-1 and NOX-4 expression, decreased mitochondrial ROS generation, and downregulated adhesion molecules such as ICAM-1 and VCAM-1 [60]. It also suppressed several pro-inflammatory mediators, including IL-1α, IL-1β, IL-6, CXCL-1, CXCL-2, and MCP-1 [59,60]. These findings suggest potential endothelial-protective effects; however, current evidence remains limited to *in vitro* and in silico studies, and further *in vivo* and clinical validation is required.

#### 4.5.3. Gypenosides

Gypenosides are triterpenoid saponins isolated from *Gynostemma pentaphyllum*. Several derivatives, including Gypenoside XLIII and LVI, have been identified as potential non-peptide PCSK9 inhibitors. Molecular docking studies suggest that gypenosides may interact with the catalytic domain of PCSK9 through hydrogen-bond interactions involving residues such as Arg-458 [61]. In hepatocyte models, gypenosides at 5 and 20 µM for 24 h reduced *PCSK9* expression by approximately 30–55% and increased LDLR protein levels by 1.5–2.0-fold, supporting their potential role in enhancing LDL-C clearance [62]. Gypenosides also reduce protein PCSK9 in statin treated hepatocyte models [62]. In addition to their lipid-regulating effects, gypenosides may also exert vascular protective activity. Experimental studies indicate that they reduce the expression of endothelial inflammatory markers, including ICAM-1, VCAM-1, and MCP-1, with reductions ranging from approximately 30% to 60% [61,63]. These effects appear to involve modulation of the PCSK9/LOX-1 signaling pathway in endothelial cells. Overall, gypenosides may contribute to both LDLR preservation and endothelial protection, although available evidence remains preclinical and requires further validation in human studies.

## 5. Translational Gap and Clinical Implications

### 5.1. Current Translational Challenges

Despite considerable progress in the development of therapies targeting PCSK9, a clear translational disconnect remains between mechanistic findings derived from experimental models and their clinical relevance for vascular function. Monoclonal antibodies, including alirocumab and evolocumab, together with siRNA-based approaches such as inclisiran, have consistently demonstrated substantial efficacy in lowering LDL-C and reducing cardiovascular events in large-scale clinical trials [37,38,39]. However, their direct impact on endothelial biology is not yet fully elucidated.

One of the principal challenges lies in the mismatch between cellular targets explored in *in vitro* studies and the pharmacological sites of action observed in clinical practice. Experimental data suggest that PCSK9 may influence endothelial inflammation, oxidative stress, and the expression of adhesion molecules via mechanisms involving NF-κB activation and reactive oxygen species generation [64,65]. In contrast, currently approved therapies predominantly exert their effects at the hepatic level by lowering circulating PCSK9 concentrations, rather than directly modulating intracellular PCSK9 signaling within endothelial cells [38]. This raises an important question as to whether observed improvements in endothelial function reflect direct vascular effects or are secondary to systemic lipid reduction and diminished inflammatory burden.

An important unresolved issue is the extent to which these vascular benefits are truly independent of LDL-C lowering [66,67]. Experimental studies consistently suggest that PCSK9 may directly modulate endothelial inflammation, oxidative stress, mitochondrial dysfunction, and programmed cell death through intracellular signaling pathways. However, current clinical trials have primarily evaluated reductions in LDL-C concentrations and cardiovascular outcomes rather than endothelial-specific mechanisms [66]. Consequently, it remains difficult to determine whether the reported improvements in endothelial function reflect genuine lipid-independent effects or arise indirectly from improved lipid metabolism and reduced systemic inflammation. Studies assessing endothelial function, such as flow-mediated dilation, have yielded encouraging findings, although improvements frequently correlate with the magnitude of LDL-C reduction, making causal interpretation challenging [6,68]. Clinical trials incorporating endothelial-specific functional endpoints, microvascular assessments, and mechanistic biomarkers will be essential to establish whether PCSK9 exerts clinically relevant lipid-independent effects on endothelial biology [6,69].

In addition, a substantial proportion of mechanistic research is based on simplified *in vitro* systems that do not fully replicate the complexity of human vascular disease. Studies involving endothelial cells frequently rely on oxidized LDL stimulation or employ supraphysiological concentrations of experimental compounds, conditions that may not accurately mirror the intricate interactions between immune responses, haemodynamic forces, and metabolic factors present *in vivo* [70,71]. As a result, the translational applicability of these findings remains uncertain.

### 5.2. Clinical Evidence and Remaining Knowledge Gaps

A further limitation is the relative lack of clinical endpoints specifically designed to evaluate endothelial function in trials of PCSK9-targeted therapies. Although reductions in circulating inflammatory markers have been documented, direct functional assessments—such as flow-mediated dilation (FMD) or measures of endothelial-dependent vasoreactivity—are seldom incorporated into large-scale outcome studies [37,38,72]. This restricts the ability to establish a clear causal link between PCSK9 inhibition and improvements in vascular function.

Emerging therapeutic approaches, including strategies aimed at intracellular protein degradation and the use of bioactive natural compounds, present promising opportunities to directly target endothelial PCSK9 signaling. However, these approaches remain largely confined to *in vitro* and early preclinical investigations, with limited *in vivo* validation and no established clinical application to date. Bridging this gap will require integrated approaches that combine advanced animal models, multi-omics technologies, and rigorously designed clinical trials incorporating vascular-specific endpoints.

In light of these challenges, future research should prioritize integrative translational strategies capable of bridging mechanistic discoveries with clinically meaningful vascular outcomes. Beyond the identification of endothelial-specific biomarkers, future investigations should incorporate advanced molecular profiling approaches, including transcriptomics, proteomics, metabolomics, and single-cell sequencing, to characterize endothelial-specific responses to PCSK9 modulation. Equally important is the development of endothelial-targeted drug delivery systems capable of selectively modulating intracellular PCSK9 signaling while minimizing systemic effects. Future clinical trials should also incorporate validated endothelial functional assessments, such as flow-mediated dilation, microvascular reactivity, vascular imaging, and circulating biomarkers of endothelial activation, to distinguish direct endothelial effects from improvements secondary to LDL-C reduction. Collectively, these multidisciplinary approaches will be essential for validating the proposed lipid-independent mechanisms of PCSK9 in humans and for translating experimental discoveries into precision therapeutic strategies targeting endothelial dysfunction.

## 6. Conclusions

In summary, PCSK9 has emerged as a complex and potentially multifunctional regulator of cardiovascular disease, extending beyond its traditional role in lipid metabolism to include possible associations with endothelial function, inflammatory processes, and oxidative stress. A growing body of evidence from *in vitro* and preclinical studies suggests that PCSK9 may contribute to endothelial dysfunction through the regulation of key signalling pathways, notably those involving NF-κB activation, reactive oxygen species production, and the expression of adhesion molecules. Collectively, these observations support PCSK9 as a plausible mechanistic link between dyslipidemia and vascular inflammation, rather than establishing it as a definitive causal mediator.

Therapies that have achieved clinical validation, particularly monoclonal antibodies and siRNA-based agents, have demonstrated marked effectiveness in lowering LDL cholesterol and reducing cardiovascular risk. However, their actions are predominantly systemic and hepatic, with limited capacity to directly influence intracellular PCSK9 signalling within vascular cells. Consequently, the degree to which these interventions confer endothelial-specific benefits remains uncertain.

Novel therapeutic approaches, including strategies based on intracellular protein degradation, peptide-derived inhibitors, and bioactive natural compounds, offer promising opportunities to overcome these limitations by directly targeting PCSK9 at the cellular level. In particular, naturally derived agents such as ginkgolide B and gypenosides display pleiotropic effects, combining lipid-lowering properties with anti-inflammatory and antioxidant activities. Nevertheless, these strategies are still largely supported by *in vitro* findings, with substantial gaps in *in vivo* validation and clinical applicability.

The comparative perspective outlined in this review highlights a clear distinction between well-established clinical therapies and emerging, mechanistically innovative approaches that remain in early stages of development. This translational divide is further exacerbated by the limited inclusion of endothelial-specific functional endpoints in clinical trials, which restricts the ability to determine causal relationships between PCSK9 inhibition and improvements in vascular function.

Moving forward, research efforts should focus on integrative strategies that bridge molecular insights with clinical outcomes. This includes the development of endothelial-targeted delivery platforms, the identification of vascular-specific biomarkers, and the incorporation of functional assessments of endothelial performance into clinical trial design. Such advances will be critical to more precisely define the therapeutic potential of PCSK9 modulation and to support the advancement of precision medicine in cardiovascular disease.

## Figures and Tables

**Table 1:** Hierarchy and Strength of Current Evidence Supporting the Proposed Mechanisms and Therapeutic Strategies of PCSK9 in Endothelial Dysfunction.

Category	Mechanism/Therapeutic Strategy	*In Vitro*	Animal Studies	Human Clinical Evidence Directly Related to Endothelial Dysfunction	Overall Strength of Evidence
Mechanistic evidence	Endothelial apoptosis	✓	✓	-	Moderate
	Endothelial pyroptosis	✓	✓	-	Moderate
	Impaired efferocytosis	✓	✓	-	Emerging
	TLR4/NF-κB signalling	✓	✓	Limited	Moderate
	LOX-1 signalling	✓	✓	Limited	Moderate
	SIRT3/SIRT4-related mitochondrial dysfunction	✓	Limited	-	Emerging
Therapeutic evidence (PCSK9 inhibitor-based studies)	Monoclonal antibodies	✓	✓	✓	High
	Inclisiran	✓	✓	✓	High
	ATTECs	✓	✓	-	Emerging
	Peptide inhibitors	✓	Limited	-	Emerging
	Natural compounds (Brazilin, Ginkgolide B, Gypenosides)	✓	Limited	-	Emerging

Evidence categories were assigned according to the highest level of currently available evidence. High indicates evidence supported by human clinical studies together with consistent preclinical findings. Moderate indicates consistent evidence derived from both *in vitro* and experimental animal studies but limited or indirect clinical validation. Emerging refers to evidence derived predominantly from mechanistic *in vitro* studies and/or early preclinical investigations, with insufficient clinical confirmation.

**Table 2:** Clinically Validated PCSK9-Targeted Therapies and Their Effects on Lipid Lowering, Cardiovascular Outcomes, and Endothelial Function.

Therapeutic Strategy	Representative Agents	Mechanism of Action	Model/Evidence Level	Major Advantages	Evidence for Endothelial Protection	Limitations
**Monoclonal Antibodies**	Alirocumab, Evolocumab	Extracellular neutralisation of circulating PCSK9, preventing LDLR degradation	Clinical (RCTs) supported by complementary *in vitro* studies	Robust LDL-C reduction; proven cardiovascular benefit	Indirect evidence through improved vascular biomarkers and reduced inflammation	No intracellular targeting; limited endothelial-specific endpoints
**siRNA**	Inclisiran	RNA-induced silencing complex (RISC)-mediated degradation of PCSK9 mRNA in hepatocytes	Clinical (Phase III) with predominantly preclinical vascular evidence	Long dosing interval; sustained PCSK9 suppression	Primarily indirect endothelial benefits via lipid lowering	Hepatocyte-specific; lack of direct endothelial evidence

**Table 3:** Emerging Experimental Strategies Targeting PCSK9: Mechanisms, Endothelial Effects, and Translational Limitations.

Therapy Class	Representative Agents	Mechanism of Action	Model/Evidence Level	Major Advantages	Endothelial Effects	Limitations
**Autophagy-based strategies**	ATTEC-like compounds	Targeted PCSK9 degradation via the autophagy–lysosome pathway	Preclinical (*in vitro*, limited *in vivo*)	Direct intracellular targeting; novel mechanism	Potential endothelial benefit through intracellular PCSK9 reduction	No validated PCSK9-specific clinical data
**Peptide inhibitors**	Pep2-8, Fusion peptides	Blockade of PCSK9–LDLR interaction	Preclinical (in vitro, limited *in vivo*)	High target specificity	Restoration of LDLR and potential improvement of endothelial efferocytosis	Poor stability, bioavailability
**Natural compounds**	Brazilin, Ginkgolide B, Gypenosides	**Brazilin** Suppression of PCSK9 expression and inflammatory signalling pathways **Ginkgolide B** Downregulation of PCSK9 and inhibition of oxidative stress pathways **Gypenosides** Decreased PCSK9 expression and increased LDLR expression	*In vitro*	**Brazilin** Multi-target activity; natural origin **Ginkgolide B** Simultaneous lipid-regulatory and anti-inflammatory effects **Gypenosides** Dual lipid-lowering and vascular protective activities	**Brazilin** Anti-inflammatory and antioxidant effects suggest endothelial protection **Ginkgolide B** Demonstrated protection against endothelial dysfunction in HUVEC models **Gypenosides** Improved endothelial function via the PCSK9/LOX-1 pathway	No clinical validation; variability & standardisation issues

## Data Availability

No new data were created or analyzed in this study. Data sharing is not applicable to this article.
